# 2-[(*E*)-2-Hy­droxy-5-(trifluoro­meth­oxy)benzyl­idene­amino]-4-methyl­phenol

**DOI:** 10.1107/S1600536810050579

**Published:** 2010-12-11

**Authors:** Aslı Tosyalı Karadağ, Şehriman Atalay, Hasan Genç

**Affiliations:** a Department of Physics, Faculty of Arts and Sciences, Ondokuz Mayıs University, Kurupelit, TR-55139 Samsun, Turkey; b Department of Chemistry, Faculty of Arts and Sciences, Yüzüncü Yıl Univercity, TR-65250 Van, Turkey

## Abstract

The title compound, C_15_H_12_F_3_NO_3_, is a Schiff base which adopts the *cis*-quinoid form in the solid state. The dihedral angle between the least-squares planes of the benzene rings being 3.6 (1)°. The F atoms of the –CF_3_ group are disordered over two sets of sites with refined occupancies of 0.61 (5) and 0.39 (5). An intra­molecular N—H⋯O hydrogen bond occurs. The crystal structure is stabilized by inter­molecular O—H⋯O hydrogen bonds.

## Related literature

Schiff base compounds can be classified by their photochromic and thermochromic characteristics, see: Calligaris *et al.* (1972 ▶); Cohen *et al.* (1964 ▶); Hadjoudis *et al.* (1987 ▶). For Schiff base tautomerism, see: Karabıyık *et al.* (2008 ▶).
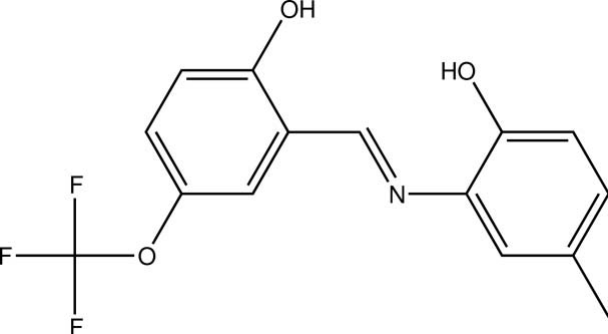


## Experimental

### 

#### Crystal data

C_15_H_12_F_3_NO_3_
*M* *_r_* = 311.26
Triclinic, 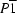

*a* = 6.4730 (5) Å
*b* = 8.4435 (6) Å
*c* = 13.0369 (9) Å
α = 82.171 (6)°
β = 88.034 (6)°
γ = 85.622 (6)°
*V* = 703.62 (9) Å^3^
*Z* = 2
Mo *K*α radiation
μ = 0.13 mm^−1^
*T* = 293 K
0.58 × 0.27 × 0.03 mm

#### Data collection

Stoe IPDS 2 diffractometer
Absorption correction: integration (*X-RED32*; Stoe & Cie, 2002 ▶) *T*_min_ = 0.953, *T*_max_ = 0.995
11152 measured reflections
2762 independent reflections
1328 reflections with *I* > 2σ(*I*)
*R*_int_ = 0.078

#### Refinement

*R*[*F* ^2^ > 2σ(*F* ^2^)] = 0.048
*wR*(*F* ^2^) = 0.077
*S* = 0.89
2762 reflections
236 parameters
3 restraints
H atoms treated by a mixture of independent and constrained refinement
Δρ_max_ = 0.10 e Å^−3^
Δρ_min_ = −0.14 e Å^−3^

### 

Data collection: *X-AREA* (Stoe & Cie, 2002 ▶); cell refinement: *X-AREA*; data reduction: *X-RED32* (Stoe & Cie, 2002 ▶); program(s) used to solve structure: *SHELXS97* (Sheldrick, 2008 ▶); program(s) used to refine structure: *SHELXL97* (Sheldrick, 2008 ▶); molecular graphics: *ORTEP-3 for Windows* (Farrugia, 1997 ▶) and *PLATON* (Spek, 2009 ▶); software used to prepare material for publication: *WinGX* (Farrugia, 1999 ▶).

## Supplementary Material

Crystal structure: contains datablocks I, global. DOI: 10.1107/S1600536810050579/zq2072sup1.cif

Structure factors: contains datablocks I. DOI: 10.1107/S1600536810050579/zq2072Isup2.hkl

Additional supplementary materials:  crystallographic information; 3D view; checkCIF report

## Figures and Tables

**Table 1:** Hydrogen-bond geometry (Å, °)

*D*—H⋯*A*	*D*—H	H⋯*A*	*D*⋯*A*	*D*—H⋯*A*
N1—H111⋯O2	0.99 (3)	1.72 (3)	2.546 (2)	138 (2)
O1—H1*A*⋯O2^i^	0.99 (3)	1.63 (3)	2.591 (2)	164 (3)

Symmetry code: (i) 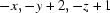.

**Fig. 1.**
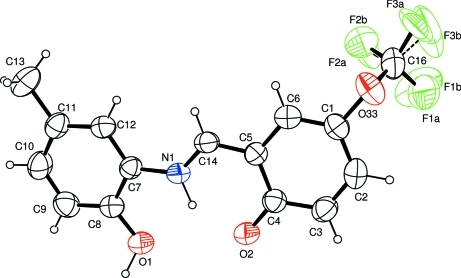
View of the molecular structure of the title compound showing the atom numbering scheme and displacement ellipsoids for the non-H atoms at the 50% probability level.

**Fig. 2.**
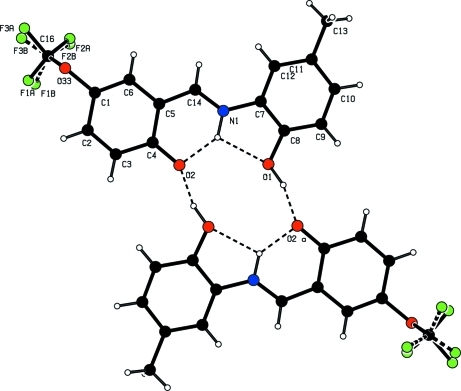
Partial packing view showing the O—H···O hydrogen bonds represented as dashed lines [symmetry code: (i)-x, -y + 2, -z + 1].

## supplementary crystallographic information

### Comment

Schiff bases have been extensively used as ligands in the field of coordination chemistry (Calligaris *et al.*, 1972). Schiff base compounds can be classified by their photochromic and thermochromic characteristics (Cohen *et al.*, 1964). These properties result from proton transfer from the hydroxyl O atom to the imine N atom (Hadjoudis *et al.*, 1987).

There are two types of intramolecular hydrogen bonds in Schiff bases, N—H···O hydrogen bond in keto-amine or N···H—O hydrogen bond in phenol-imine tautomeric forms (Karabıyık *et al.*, 2008).

The present X-ray investigation shows that the title compound is a Schiff base which exists in the *cis*-quinoid form in the solid-state. A *PLATON* plot of the molecule is shown in Fig.1. The molecule is nearly planar, the angle between the least-squares planes of the benzene rings being 3.6 (1)°. The F atoms of the CF_3_ group are disordered over two sets of sites with refined occupancies of 0.61 (5) and 0.39 (5). The N1—C14 bond length of 1.305 (3) Å is typical of a double bond. The crystal structure is stabilized by intra- and intermolecular O—H···O and N—H···O hydrogen bonds.

### Experimental

The title compound was prepared by the reaction of a solution containing 2-hydroxy-5-(trifluoromethoxy)benzaldehyde (0.045 g 0.23 mmol) in 20 ml ethanol and a solution containing 4-amino-4-methylphenol (0.029 g 0.23 mmol) in 20 ml ethanol. The reaction mixture was stirred for 1 h under reflux. Crystals of the title compound suitable for a X-ray analysis were obtained from ethylalcohol by slow evaporation (yield 64%; m.p.402–408 K).

### Refinement

The structure of the title compound was solved by direct methods and refined by full-matrix least-square techniques. The H atoms bonded to O1 and N1 were freely refined. All other H atoms were placed in calculated positions and refined using a riding model, with C—H = 0.93 Å and *U*_iso_(H) = 1.2*U*_eq_(C) for aromatic H atoms, and with C—H = 0.96 Å and *U*_iso_(H) = 1.5*U*_eq_(C) for methyl H atoms.

### Crystal data

**Table:**

C_15_H_12_F_3_NO_3_	*Z* = 2
*M**_r_* = 311.26	*F*(000) = 320
Triclinic, *P*1	*D*_x_ = 1.469 Mg m^−^^3^
Hall symbol: -P 1	Mo *K*α radiation, λ = 0.71073 Å
*a* = 6.4730 (5) Å	Cell parameters from 7349 reflections
*b* = 8.4435 (6) Å	θ = 1.6–27.9°
*c* = 13.0369 (9) Å	µ = 0.13 mm^−^^1^
α = 82.171 (6)°	*T* = 293 K
β = 88.034 (6)°	Prism, yellow
γ = 85.622 (6)°	0.58 × 0.27 × 0.03 mm
*V* = 703.62 (9) Å^3^	

### Data collection

**Table:**

Stoe IPDS 2 diffractometer	2762 independent reflections
Radiation source: fine-focus sealed tube	1328 reflections with *I* > 2σ(*I*)
graphite	*R*_int_ = 0.078
Detector resolution: 6.67 pixels mm^-1^	θ_max_ = 26.0°, θ_min_ = 1.6°
rotation method scans	*h* = −7→7
Absorption correction: integration (*X-RED32*; Stoe & Cie, 2002)	*k* = −10→10
*T*_min_ = 0.953, *T*_max_ = 0.995	*l* = −16→16
11152 measured reflections	

### Refinement

**Table:**

Refinement on *F*^2^	Primary atom site location: structure-invariant direct methods
Least-squares matrix: full	Secondary atom site location: difference Fourier map
*R*[*F*^2^ > 2σ(*F*^2^)] = 0.048	Hydrogen site location: inferred from neighbouring sites
*wR*(*F*^2^) = 0.077	H atoms treated by a mixture of independent and constrained refinement
*S* = 0.89	*w* = 1/[σ^2^(*F*_o_^2^) + (0.0202*P*)^2^] where *P* = (*F*_o_^2^ + 2*F*_c_^2^)/3
2762 reflections	(Δ/σ)_max_ = 0.001
236 parameters	Δρ_max_ = 0.10 e Å^−^^3^
3 restraints	Δρ_min_ = −0.14 e Å^−^^3^

### Special details

**Table:**

Geometry. All e.s.d.'s (except the e.s.d. in the dihedral angle between two l.s. planes) are estimated using the full covariance matrix. The cell e.s.d.'s are taken into account individually in the estimation of e.s.d.'s in distances, angles and torsion angles; correlations between e.s.d.'s in cell parameters are only used when they are defined by crystal symmetry. An approximate (isotropic) treatment of cell e.s.d.'s is used for estimating e.s.d.'s involving l.s. planes.
Refinement. Refinement of *F*^2^ against ALL reflections. The weighted *R*-factor *wR* and goodness of fit *S* are based on *F*^2^, conventional *R*-factors *R* are based on *F*, with *F* set to zero for negative *F*^2^. The threshold expression of *F*^2^ > σ(*F*^2^) is used only for calculating *R*-factors(gt) *etc*. and is not relevant to the choice of reflections for refinement. *R*-factors based on *F*^2^ are statistically about twice as large as those based on *F*, and *R*- factors based on ALL data will be even larger.

### Fractional atomic coordinates and isotropic or equivalent isotropic displacement parameters (Å^2^)

**Table:**

	*x*	*y*	*z*	*U*_iso_*/*U*_eq_	Occ. (<1)
C16	0.9623 (6)	0.7727 (5)	0.9480 (2)	0.0728 (9)	
H1A	−0.054 (5)	0.899 (4)	0.399 (2)	0.114 (12)*	
H111	0.303 (4)	0.857 (3)	0.540 (2)	0.097 (10)*	
C1	0.7674 (4)	0.9053 (3)	0.81076 (18)	0.0484 (6)	
C2	0.5942 (4)	0.9960 (3)	0.84348 (18)	0.0561 (7)	
H2	0.5980	1.0412	0.9045	0.067*	
C3	0.4201 (4)	1.0184 (3)	0.78629 (18)	0.0544 (7)	
H3	0.3059	1.0792	0.8089	0.065*	
C4	0.4088 (4)	0.9514 (3)	0.69342 (17)	0.0462 (6)	
C5	0.5886 (4)	0.8589 (3)	0.66153 (16)	0.0427 (6)	
C6	0.7678 (4)	0.8403 (3)	0.72199 (18)	0.0486 (6)	
H6	0.8861	0.7830	0.7005	0.058*	
C7	0.3895 (4)	0.7180 (3)	0.42719 (17)	0.0424 (6)	
C8	0.1917 (4)	0.7462 (3)	0.38710 (18)	0.0477 (6)	
C9	0.1518 (4)	0.6878 (3)	0.29666 (19)	0.0592 (7)	
H9	0.0212	0.7074	0.2680	0.071*	
C10	0.3056 (4)	0.6007 (3)	0.2487 (2)	0.0602 (8)	
H10	0.2760	0.5618	0.1879	0.072*	
C11	0.5026 (4)	0.5689 (3)	0.28793 (18)	0.0500 (6)	
C12	0.5427 (4)	0.6285 (3)	0.37817 (17)	0.0464 (6)	
H12	0.6736	0.6087	0.4065	0.056*	
C13	0.6709 (4)	0.4766 (3)	0.2318 (2)	0.0702 (8)	
H13A	0.7443	0.5501	0.1838	0.105*	
H13B	0.6093	0.4019	0.1950	0.105*	
H13C	0.7657	0.4194	0.2812	0.105*	
C14	0.5861 (4)	0.7821 (3)	0.57199 (17)	0.0442 (6)	
H14	0.7059	0.7253	0.5516	0.053*	
F1A	0.8285 (13)	0.808 (3)	1.0198 (5)	0.120 (4)	0.61 (5)
F2B	0.952 (2)	0.6343 (7)	0.9169 (9)	0.106 (3)	0.61 (5)
F3A	1.1534 (11)	0.7647 (17)	0.9796 (12)	0.103 (3)	0.61 (5)
F1B	0.819 (2)	0.769 (3)	1.0204 (10)	0.141 (7)	0.39 (5)
F2A	0.916 (4)	0.6340 (8)	0.9273 (14)	0.110 (6)	0.39 (5)
F3B	1.122 (4)	0.771 (3)	1.007 (3)	0.154 (8)	0.39 (5)
N1	0.4201 (3)	0.7886 (2)	0.51702 (14)	0.0433 (5)	
O1	0.0485 (3)	0.8293 (2)	0.44187 (13)	0.0619 (5)	
O2	0.2431 (2)	0.9701 (2)	0.63948 (12)	0.0576 (5)	
O33	0.9507 (3)	0.8897 (2)	0.86952 (14)	0.0682 (6)	

### Atomic displacement parameters (Å^2^)

**Table:**

	*U*^11^	*U*^22^	*U*^33^	*U*^12^	*U*^13^	*U*^23^
C16	0.093 (3)	0.080 (3)	0.0477 (19)	0.007 (2)	−0.0135 (18)	−0.0181 (19)
C1	0.0470 (17)	0.0509 (17)	0.0475 (14)	−0.0074 (14)	−0.0058 (12)	−0.0040 (13)
C2	0.0695 (19)	0.0587 (18)	0.0424 (14)	−0.0121 (15)	0.0007 (13)	−0.0112 (13)
C3	0.0542 (17)	0.0556 (18)	0.0531 (15)	0.0019 (14)	0.0066 (13)	−0.0113 (13)
C4	0.0469 (16)	0.0454 (16)	0.0456 (14)	−0.0011 (13)	−0.0006 (12)	−0.0048 (12)
C5	0.0419 (16)	0.0447 (16)	0.0411 (13)	−0.0005 (13)	0.0000 (12)	−0.0060 (12)
C6	0.0441 (16)	0.0467 (16)	0.0540 (15)	−0.0001 (12)	−0.0006 (12)	−0.0049 (13)
C7	0.0465 (17)	0.0377 (15)	0.0430 (14)	−0.0025 (13)	0.0011 (12)	−0.0060 (11)
C8	0.0465 (16)	0.0452 (17)	0.0508 (14)	−0.0001 (13)	−0.0013 (12)	−0.0054 (13)
C9	0.0595 (19)	0.0566 (18)	0.0634 (17)	−0.0042 (15)	−0.0145 (14)	−0.0108 (14)
C10	0.072 (2)	0.0552 (19)	0.0583 (16)	−0.0090 (16)	−0.0038 (15)	−0.0207 (14)
C11	0.0616 (18)	0.0391 (16)	0.0499 (14)	−0.0065 (13)	0.0073 (12)	−0.0089 (12)
C12	0.0453 (16)	0.0418 (16)	0.0510 (15)	0.0005 (13)	0.0001 (12)	−0.0042 (12)
C13	0.080 (2)	0.0564 (18)	0.0773 (19)	−0.0037 (16)	0.0181 (16)	−0.0256 (15)
C14	0.0404 (16)	0.0428 (16)	0.0470 (14)	0.0040 (12)	0.0026 (12)	−0.0023 (12)
F1A	0.153 (7)	0.149 (9)	0.046 (4)	0.051 (4)	0.002 (4)	−0.007 (4)
F2B	0.127 (5)	0.085 (7)	0.106 (5)	0.031 (5)	−0.024 (4)	−0.030 (5)
F3A	0.069 (6)	0.158 (5)	0.078 (5)	0.007 (3)	−0.048 (3)	−0.003 (4)
F1B	0.190 (14)	0.108 (9)	0.098 (10)	0.030 (6)	0.077 (11)	0.037 (7)
F2A	0.189 (14)	0.045 (7)	0.094 (8)	−0.013 (7)	−0.087 (9)	0.019 (6)
F3B	0.22 (2)	0.165 (10)	0.082 (11)	−0.021 (10)	−0.082 (9)	−0.007 (7)
N1	0.0387 (13)	0.0436 (14)	0.0467 (12)	0.0048 (10)	−0.0011 (10)	−0.0063 (10)
O1	0.0488 (11)	0.0748 (14)	0.0604 (11)	0.0158 (10)	−0.0036 (9)	−0.0138 (10)
O2	0.0461 (11)	0.0673 (13)	0.0592 (10)	0.0141 (9)	−0.0051 (9)	−0.0173 (9)
O33	0.0648 (14)	0.0784 (14)	0.0620 (12)	−0.0158 (11)	−0.0203 (10)	−0.0008 (11)

### Geometric parameters (Å, °)

**Table:**

C16—F2B	1.294 (5)	C7—C8	1.391 (3)
C16—F2A	1.295 (5)	C7—C12	1.392 (3)
C16—F1B	1.302 (6)	C7—N1	1.411 (3)
C16—F1A	1.303 (5)	C8—O1	1.362 (3)
C16—F3A	1.312 (5)	C8—C9	1.378 (3)
C16—F3B	1.312 (6)	C9—C10	1.376 (3)
C16—O33	1.322 (3)	C9—H9	0.9300
C1—C6	1.347 (3)	C10—C11	1.383 (3)
C1—C2	1.396 (3)	C10—H10	0.9300
C1—O33	1.422 (3)	C11—C12	1.381 (3)
C2—C3	1.360 (3)	C11—C13	1.517 (3)
C2—H2	0.9300	C12—H12	0.9300
C3—C4	1.411 (3)	C13—H13A	0.9600
C3—H3	0.9300	C13—H13B	0.9600
C4—O2	1.291 (3)	C13—H13C	0.9600
C4—C5	1.433 (3)	C14—N1	1.305 (3)
C5—C6	1.411 (3)	C14—H14	0.9300
C5—C14	1.412 (3)	N1—H111	0.98 (3)
C6—H6	0.9300	O1—H1A	0.99 (3)
			
F2B—C16—F1B	101.8 (16)	C8—C7—N1	115.3 (2)
F2A—C16—F1A	105 (2)	C12—C7—N1	124.3 (2)
F2A—C16—F3A	110.3 (14)	O1—C8—C9	124.3 (2)
F1A—C16—F3A	111.9 (7)	O1—C8—C7	116.8 (2)
F2B—C16—F3B	110.6 (14)	C9—C8—C7	118.9 (2)
F1B—C16—F3B	97.7 (19)	C10—C9—C8	119.9 (2)
F2B—C16—O33	111.3 (5)	C10—C9—H9	120.1
F2A—C16—O33	115.5 (7)	C8—C9—H9	120.1
F1B—C16—O33	119.0 (11)	C9—C10—C11	122.3 (2)
F1A—C16—O33	108.7 (9)	C9—C10—H10	118.8
F3A—C16—O33	105.6 (7)	C11—C10—H10	118.8
F3B—C16—O33	115.1 (14)	C12—C11—C10	117.7 (2)
C6—C1—C2	121.3 (2)	C12—C11—C13	121.1 (2)
C6—C1—O33	119.5 (2)	C10—C11—C13	121.2 (2)
C2—C1—O33	119.0 (2)	C11—C12—C7	120.7 (2)
C3—C2—C1	120.1 (2)	C11—C12—H12	119.6
C3—C2—H2	119.9	C7—C12—H12	119.6
C1—C2—H2	119.9	C11—C13—H13A	109.5
C2—C3—C4	121.4 (2)	C11—C13—H13B	109.5
C2—C3—H3	119.3	H13A—C13—H13B	109.5
C4—C3—H3	119.3	C11—C13—H13C	109.5
O2—C4—C3	121.8 (2)	H13A—C13—H13C	109.5
O2—C4—C5	120.8 (2)	H13B—C13—H13C	109.5
C3—C4—C5	117.3 (2)	N1—C14—C5	121.7 (2)
C6—C5—C14	119.7 (2)	N1—C14—H14	119.1
C6—C5—C4	119.7 (2)	C5—C14—H14	119.1
C14—C5—C4	120.6 (2)	C14—N1—C7	129.2 (2)
C1—C6—C5	120.1 (2)	C14—N1—H111	114.7 (17)
C1—C6—H6	120.0	C7—N1—H111	116.0 (17)
C5—C6—H6	120.0	C8—O1—H1A	114.9 (17)
C8—C7—C12	120.4 (2)	C16—O33—C1	116.4 (2)
			
C6—C1—C2—C3	0.9 (4)	C9—C10—C11—C12	−0.3 (4)
O33—C1—C2—C3	177.2 (2)	C9—C10—C11—C13	−178.2 (3)
C1—C2—C3—C4	0.1 (4)	C10—C11—C12—C7	−0.3 (3)
C2—C3—C4—O2	179.0 (3)	C13—C11—C12—C7	177.7 (3)
C2—C3—C4—C5	−0.1 (4)	C8—C7—C12—C11	1.3 (4)
O2—C4—C5—C6	−180.0 (2)	N1—C7—C12—C11	−177.3 (2)
C3—C4—C5—C6	−0.9 (3)	C6—C5—C14—N1	176.1 (2)
O2—C4—C5—C14	−1.9 (4)	C4—C5—C14—N1	−1.9 (3)
C3—C4—C5—C14	177.2 (2)	C5—C14—N1—C7	−179.0 (2)
C2—C1—C6—C5	−2.0 (4)	C8—C7—N1—C14	179.2 (3)
O33—C1—C6—C5	−178.2 (2)	C12—C7—N1—C14	−2.1 (4)
C14—C5—C6—C1	−176.1 (2)	F2B—C16—O33—C1	60.1 (7)
C4—C5—C6—C1	1.9 (4)	F2A—C16—O33—C1	48.0 (16)
C12—C7—C8—O1	177.5 (2)	F1B—C16—O33—C1	−57.8 (14)
N1—C7—C8—O1	−3.8 (3)	F1A—C16—O33—C1	−69.6 (9)
C12—C7—C8—C9	−1.8 (4)	F3A—C16—O33—C1	170.2 (7)
N1—C7—C8—C9	176.9 (2)	F3B—C16—O33—C1	−173.1 (19)
O1—C8—C9—C10	−177.9 (3)	C6—C1—O33—C16	−97.4 (3)
C7—C8—C9—C10	1.3 (4)	C2—C1—O33—C16	86.3 (3)
C8—C9—C10—C11	−0.2 (4)		

### Hydrogen-bond geometry (Å, °)

**Table:**

*D*—H···*A*	*D*—H	H···*A*	*D*···*A*	*D*—H···*A*
N1—H111···O1	0.99 (3)	2.16 (3)	2.610 (3)	106 (2)
N1—H111···O2	0.99 (3)	1.72 (3)	2.546 (2)	138 (2)
O1—H1A···O2^i^	0.99 (3)	1.63 (3)	2.591 (2)	164 (3)

Symmetry codes: (i) −*x*, −*y*+2, −*z*+1.
